# Investigation of Attenuation Correction for Small-Animal Single Photon Emission Computed Tomography

**DOI:** 10.1155/2013/430276

**Published:** 2013-06-11

**Authors:** Hsin-Hui Lee, Jyh-Cheng Chen

**Affiliations:** Department of Biomedical Imaging and Radiological Sciences, National Yang-Ming University, Experimental Building B306, No. 155, Section 2, Li-Nong Street, Baitou, Taipei City 112, Taiwan

## Abstract

The quantitative accuracy of SPECT is limited by photon attenuation and scatter effect when photons interact with atoms. In this study, we developed a new attenuation correction (AC) method, CT-based mean attenuation correction (CTMAC) method, and compared it with various methods that were often used currently to assess the AC phenomenon by using the small-animal SPECT/CT data that were acquired from various physical phantoms and a rat. The physical phantoms and an SD rat, which were injected with ^99m^Tc, were scanned by a parallel-hole small-animal SPECT, and then they were imaged by the 80 kVp micro-CT. Scatter was estimated and corrected by the triple-energy window (TEW) method. Absolute quantification was derived from a known activity point source scan. In the physical-phantom studies, we compared the images with original, scatter correction (SC) only, and the scatter-corrected images with AC performed by using Chang's method, CT-based attenuation correction (CTAC), CT-based iterative attenuation compensation during reconstruction (CTIACR), and the CTMAC. From the correction results, we find out that the errors of the previous six configurations are mostly quite similar. The CTMAC needs the shortest correction time while obtaining good AC results.

## 1. Introduction

In order to improve the image quality of SPECT in clinical practice and the quantitative accuracy, photon attenuation correction (AC) has been broadly studied. It has been applied in territories, such as cardiac imaging, brain functional imaging, and cancer imaging. Attenuation correction is indispensible especially when we need accurate quantification of radioisotope concentration [1–13]. 

The photon energy emitted from the radioisotopes used for SPECT is mostly less than 400 keV. In soft tissues, Compton scattering effect is the dominant interaction for energy above 50 keV, followed by photoelectric effect. Attenuation is the phenomenon that the intensity of radiation decreases due to the photons scattered or absorbed by materials when the radiation goes through materials. There are three factors affecting photon attenuation. Firstly, it is energy, which is the characteristic of the radiation itself. The photon of higher energy is not easier to be attenuated and is easier to penetrate materials. The other two are related to materials; they are density and atomic number, respectively, and the probability of interacting with photons increases when the values are larger [14]. The methods most used to get attenuation map are to be measured by CT [9, 15–17] or to perform transmission computed tomography by gamma ray of radionuclide [11, 12, 18]. But currently, there are no small-animal SPECT scanners equipped with transmission source of radionuclide yet although there is need for AC in rat imaging to produce quantitative SPECT.

The center of our study focuses on correcting the effect of photon attenuation, to let the SPECT of FLEX Triumph preclinical imaging system (Gamma Medica-Ideas, Northridge, CA) be capable of providing quantitative information. The images obtained by hybrid SPECT/CT system are legible, and quantitative accuracy of imaging results can be improved as the anatomy information provided by CT can clearly and accurately depict SPECT images, and CT images can also provide the information of photon attenuation inside of materials [19, 20].

When Seo et al. [21] performed imaging with ^111^In which emit gamma rays at 171 keV (94%) and 245 keV (90.2%) in the decay processes, the authors calculated the effective gamma ray energy with the concept of average energy which is 210 keV. The linear calibration curve was used to convert CT numbers to nonuniform attenuation maps of 210 keV gamma rays related to materials. These nonuniform attenuation maps were used to perform attenuation correction with SPECT data. The concept of utilizing average effective values to correct imaging data was applied to our method.

We evaluate the advantages and disadvantages of several attenuation correction methods in this paper through the corrected results of the imaging data of different physical phantoms and animal experiments by using several attenuation correction methods that are more commonly used currently and our method on the same condition that they were combined with triple-energy window (TEW) method for scatter correction (SC) [22]. In image domain, we used Chang's method and CT-based attenuation correction (CTAC) to perform attenuation corrections with the images reconstructed by filtered backprojection (FBP). During reconstruction, we used iterative algorithms combined with the system matrix, which contains the probability of photon penetration, to reconstruct images. When image reconstruction is completed, photon attenuation effect is also compensated. In Radon space, we used our AC method to perform attenuation correction on measured projection data and then combined with FBP to reconstruct images; in this way we can obtain attenuation and scatter corrected SPECT images. Radon space is a 2D dataset *p*(*r*, *θ*) obtained by stacking the 1D projections, where *r* corresponds to different positions of detector element, and *θ* corresponds to the scanning angles of SPECT.

The contribution of our study is that we compared various AC methods which were often used currently with our CTMAC method to perform attenuation correction based on the animal SPECT with the parallel-hole collimator and then analyzed the results after AC and investigated the advantages and disadvantages of each method; the trustworthiness of quantitative result after scanning can be enhanced in this way.

## 2. Materials and Methods

### 2.1. Small-Animal SPECT Description

The gamma camera is consisted of 5 by 5 solid cadmium zinc telluride (CZT) modules. Each module consists of 16 by 16 pixels. The pixel size is 1.5 mm. Thus, each gamma camera contains 80 by 80 pixels. The FOV size is 12.7 cm by 12.7 cm. The sensitivity of 1 camera can reach 1000 cps/MBq/mL. Because it belongs to a semiconductor detector, the energy resolution is excellent, about 4.6%, and therefore can effectively distinguish between photopeak and scattered photons whose energies are close to photopeak; two photopeaks that have similar energy can be identified also. Take CZT detector in 2 mm thickness as example; the stopping efficiency for 140 keV gamma rays is about 40% [23]. The FLEX Triumph preclinical X-SPECT subsystem installed in Taipei Veteran General Hospital is equipped with three gamma camera heads. During the imaging process, each detector was equipped with a high resolution parallel-hole collimator. The aperture size is 1.2 mm. Each gamma camera head scans 180° (actually scans 174.375°), the head 1 gamma camera scans from 0° to 180°, the head 2 gamma camera scans from 180° to 360°, and the head 3 gamma camera scans from 90° to 270°, respectively. The radius of rotation is 120 mm.

### 2.2. Micro-CT Description

The detector of micro-CT subsystem is configured with a CMOS X-ray detector coupled to a CsI scintillation crystal with resolution of 8 line pairs/mm. The operational range of tube voltage for the internal air-cooled X-ray tube is from 40 kVp to 80 kVp. The focal spot size is 75 *μ*m. Total power is 40 W. The fixed anode target of CT is made of tungsten with cone angle of 38°. The matrix size is 2240 × 2368. Each pixel size is 50 *μ*m.

### 2.3. Image Registration and Fusion

We use Amira (Visualization Sciences Group, Burlington, USA) to perform registration and fusion of SPECT/CT images, which is a powerful, multifaceted 3D postprocessing and analysis software. CT images must be downsampled to match the matrix size of SPECT images. After that, we smoothed CT images with a suitable low-pass filter to approach the spatial resolution of SPECT images; meanwhile, noise and artifacts of CT images can be effectively reduced [2].

### 2.4. Attenuation Map

Each pixel of reconstructed images represents attenuation coefficient of objects at that point and the value usually expressed in CT number and the unit HU (Hounsfield units) defined as follows:

(1) $$\begin{array}{c} \text{CT}=\frac{\mu -\mu_{w}}{\mu_{w}}\times K, \end{array}$$

where *μ* and *μ*_*w*_ are the linear attenuation coefficient of object at a position and water, respectively, and *K* value is usually 1000. CT number represents attenuation coefficient of that tissue relative to water.

The nonuniform attenuation map which is used to correct SPECT data now is converted through linear calibration curve [2, 16, 24], but in this study, the linear attenuation coefficients of the specific photon energy in materials are converted through bilinear calibration curve by CT number measured by X-ray beams in CT imaging and the charts and data provided by the website of the physics measurement laboratory of National Institute of Standards and Technology, NIST [25, 26], which as if the method now in common use of correcting the positron emission tomography (PET) data [15, 27].

The vertical axis of bilinear calibration curve is the linear attenuation coefficient of 140 keV gamma ray emitted from ^99m^Tc in air, water, and cortical bone which are obtained by looking up tables; as for the CT number of horizontal axis, the CT number of cortical bone is obtained by choosing from the mean value of CT number of cortical bone of the left front leg of a rat, and the CT numbers of air and water are set to −1000 HU and 0 HU, respectively, so we can obtain the following functions:

(2) $$\begin{array}{c} \mu \left({\text{cm}}^{-1}\right)=0.00015454\times I\left(\text{HU}\right)+0.15454\;\forall \;\text{HU}≦0,\\ \mu \left({\text{cm}}^{-1}\right)=0.000087004\times I\left(\text{HU}\right)+0.15454\;\forall \;\text{HU}>0. \end{array}$$

### 2.5. Scatter Correction

**Figure 1:**
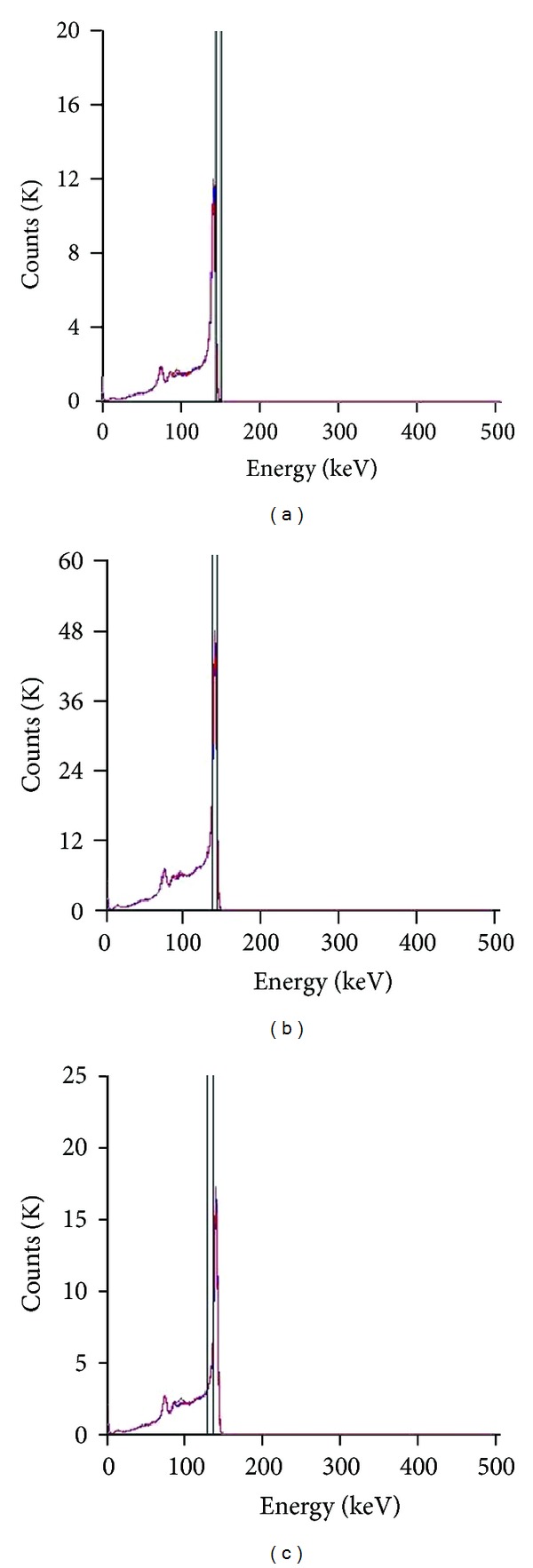
Energy spectrum of ^99m^Tc measured by CZT semiconductor gamma camera during imaging acquisition and the position and widths of the main energy window and the two subwindows with TEW method.

We used TEW method to perform the scatter correction (SC) [22, 28, 29] with the measured projection data obtained from physical phantoms and animal experiments; see Figure 1. The main energy window targets at the photopeak and opens 5% wide (130 keV to 144 keV). Two subwindows also open 5% wide. The opening range of the low subwindow is from 130 keV to 137 keV. The opening range of the high subwindow is from 144 keV to 151 keV.

### 2.6. Decay Correction

Because the acquisition time for each projection angle of SPECT is fixed, we can calculate the remaining percentage after decay through Beer's law, the reciprocal of the percentage multiplied by the measured projection data, and that can compensate the effect of decay upon the acquisition data. The same projection angle of different slices is multiplied by the same decay correction factor. This study combines different attenuation correction methods with this decay correction method to correct isotope decay.

### 2.7. Attenuation Correction

The attenuation correction factors of Chang's method are calculated pixel by pixel; therefore, it is not necessary to generate a new system matrix. Before attenuation correction performed by Chang's method [11, 30], we use FBP to perform image reconstructions on decay and scatter corrected SPECT projection data. Attenuation maps were obtained by the contours information of physical phantoms and small animal by CT [21] and then combined with Otsu's method [31] to distinguish between inside and outside of objects; assume that the linear attenuation coefficients of tissues inside of the contour are the same, which are all equal to the linear attenuation coefficient of water at 140 keV gamma rays emitted from ^99m^Tc. Consider

(3) $$\begin{array}{c} \text{ACF}\left(x,y\right)=\frac{1}{(1/N)\sum_{i=1}^{N}e^{-\mu x_{i}}}, \end{array}$$

where *μ* is the assumed linear attenuation coefficient, and *x*_*i*_ is the length of attenuation path for the pixel (*x*, *y*) at projection angle *i*. *N* = 64 is the photon attenuation path number.

The attenuation correction factors of CTAC obtained from the individual calculations at different pixel positions are the same as Chang's method. Attenuation maps are acquired through converting micro-CT images to linear attenuation coefficients at 140 keV photons for different materials by bilinear calibration curve. The attenuation correction factors at different pixel positions can be acquired by

(4) $$\begin{array}{c} \text{ACF}\left(x,y\right)=\frac{1}{(1/N)\sum_{i=1}^{N}e^{-\sum_{b}\mu_{ib}\Delta x_{ib}}}, \end{array}$$

where *N* = 64 is the photon attenuation path number. *μ*_*ib*_ is the linear attenuation coefficient for the pixel position (*x*, *y*) at sampling point *b* of projection angle *i*. Δ*x*_*ib*_ is the length of attenuation path between sampling point *b* and sampling point *b* − 1. We sampled once per unit length.

In every iterative process of CT-based iterative attenuation compensation during reconstruction (CTIACR) [1–3, 21, 28, 32, 33], there were more or less differences between the projection data of guess images and the projection data that have been corrected for scatter and decay. The system matrix used in the projection process contains the effect of photon attenuation, and we calculate the probability of photons incident on gamma cameras that are emitted from different pixel positions (*x*, *y*) towards different projection angles (*θ*). We consider the influence of different length of attenuation path of photons (the distance of the pixel position of emission to the detector surface) and the attenuation path passing through various attenuation materials at different projection angles by the attenuation map obtained from CT and then consider the concept of ray-tracing, that is; the probability of gamma photons emitted from different pixel positions that are received by different detector elements of different gamma cameras at different projection angles. Thus the error image, after comparing the last reconstructed guess image, can be obtained, so we can obtain a newer guess image. This process generally needs to be repeated several times until an estimated image, which is close to our expectation, is reached. The nonuniform attenuation maps obtained from CT can perform attenuation correction to any object. However, when the count in projection data is inadequate, the iterative process apparently enhances the statistical noise. In order to accelerate the reconstruction process, we use ordered-subsets expectation maximization (OSEM) algorithm [34] to reconstruct images. The advantage of this method is that it can model several physical phenomena easily into the system matrix, but in this study, there is no other attenuation correction method to use system matrix except for the CTIACR, and scatter and decay were unified to correct on the measured projection data, and there is only attenuation effect mounted into the system matrix. The parameters of image reconstruction for the rat-sized phantom are 2 iterations with 8 subsets. The four-quarter phantom is 5 iterations with 4 subsets. The concentric phantom is 3 iterations with 8 subsets. The myocardial perfusion imaging in an SD rat is 2 iterations with 4 subsets to acquire the reconstructed images that have been compensated for attenuation.

When gamma camera measures at one projection angle, the measured counts at one detector element are summed up of gamma photons emitted from different positions on the line of response (LOR), and the number of gamma photons contributed from these positions is different. The length of attenuation path and the linear attenuation coefficients of various materials these gamma photons passed were not the same, and we cannot obtain the information given earlier if we only have the measured projection data. Because we cannot know the real activity concentration at any pixel position on the LOR, the number of gamma photons emitted from any pixel position to detector is also unknown. CT-based mean attenuation correction (CTMAC) directly performs attenuation correction in Radon space on the measured projection data that have been corrected for scatter and decay by calculating the average of the sum of the linear attenuation coefficients at different pixel positions on the LOR and the average length of attenuation path that gamma rays passed on the LOR. Each collimator hole views the radioactivity within a cylinder perpendicular to the face of the gamma camera called its line of response (LOR). Certainly the average *μ* obtained from calculations for the LOR may not be exactly equal to the linear attenuation coefficient at any pixel position on the LOR, such as the method of Seo et al. [21]; the 210 keV gamma rays are absolutely not emitted in the decay process of ^111^In, it is just an average effective value, and it does not actually exist. This is the concept of using average effective values to correct imaging data. The results corrected by this method for any detector element at different projection angles are average cumulative photon counts, which may be more or less than the ideal value, and if you only watch the corrected result of any angle, it may be wrong. However, when the scanning angles are dense enough and the angular range covered by imaging is large enough by gamma cameras, the results reconstructed by FBP will be pretty close to the distribution of true activity concentrations. The correction process of CTMAC was performed slice by slice. The attenuation correction factor matrix corresponding to the projection data of any slice can be expressed in the following function:

(5) $$\begin{array}{c} \text{ACF}\left(r,\theta \right)=e^{\left[\int_{-\infty }^{\infty }\mu (\xi ,\eta )d\eta /\int_{-\infty }^{\infty }pl(\xi ,\eta )d\eta ]\times [\left(\int_{-\infty }^{\infty }pl(\xi ,\eta )d\eta -1\text{pixsize}\right)/2\right]}. \end{array}$$

The vertical axis *r* corresponds to different positions of detector element, the horizontal axis *θ* corresponds to the scanning angles of SPECT, *ξ* = *x*cos⁡*θ* + *y*sin*θ*, and *η* = −*x*sin*θ* + *y*cos⁡*θ*. The rotation coordinate system of CT (*ξ*, *η*) is the same as the rotation coordinate system of SPECT (*r*, *s*) because the imaging data of two systems have been registered and fused before corrections, and two different symbols sets are used for distinguishing two different imaging systems. ∫_−*∞*_^*∞*^ *μ*(*ξ*, *η*)*dη* is the projection data that the nonuniform attenuation map obtained from CT-based transformation integrated along *η* direction. ∫_−*∞*_^*∞*^ *pl*(*ξ*, *η*)*dη* is the projection data that the uniform matrix *pl*(*ξ*, *η*) (all filled with one) was integrated along *η* direction. 1 pixsize represents the pixel size of SPECT, 0.15 cm. The sinogram obtained from ∫_−*∞*_^*∞*^ *pl*(*ξ*, *η*)*dη* represents the pixels lengths included on LOR of any detector element at different projection angles. Subtracting 1 is because the point source itself smaller than or equal to one pixel size almost would not cause photon attenuation (pixel size: 1.5 × 1.5 mm^2^). That is, when any detector element of the gamma camera locates at a certain projection angle, the gamma rays emitted from the closest pixel position toward the direction of this detector element are not blocked from the attenuator of other pixel positions on the LOR, and the lengths of passed attenuation paths are insufficient for one pixel length before receiving by the gamma camera. Therefore, the average length of attenuation path of gamma rays emitted from the pixel position closest to the gamma camera is set as 0. Thus when gamma rays emitted from *N* different pixel positions on the LOR before receiving by the gamma camera, the average length of attenuation path gamma rays passed can be expressed in the following function:

(6) [0+(∫−∞∞pl(ξ,η)dη−1pixsize)]×N2×1N  =∫−∞∞pl(ξ,η)dη−1pixsize2.

∫_−*∞*_^*∞*^ *μ*(*ξ*, *η*)*dη*/∫_−*∞*_^*∞*^ *pl*(*ξ*, *η*)*dη* describes the average linear attenuation coefficients of any detector element at different projection angles on the LOR. The denominator of ∫_−*∞*_^*∞*^ *μ*(*ξ*, *η*)*dη*/∫_−*∞*_^*∞*^ *pl*(*ξ*, *η*)*dη* and the numerator of [∫_−*∞*_^*∞*^ *pl*(*ξ*, *η*)*dη* − 1pixsize]/2 are very close, although the correction process is still calculated with formula (5); formula (5) can be simplified by reducing a fraction as follows:

(7) $$\begin{array}{c} \text{ACF}\left(r,\theta \right)=e^{(\int_{-\infty }^{\infty }\mu (\xi ,\eta )d\eta )/2}. \end{array}$$

**Figure 2:**
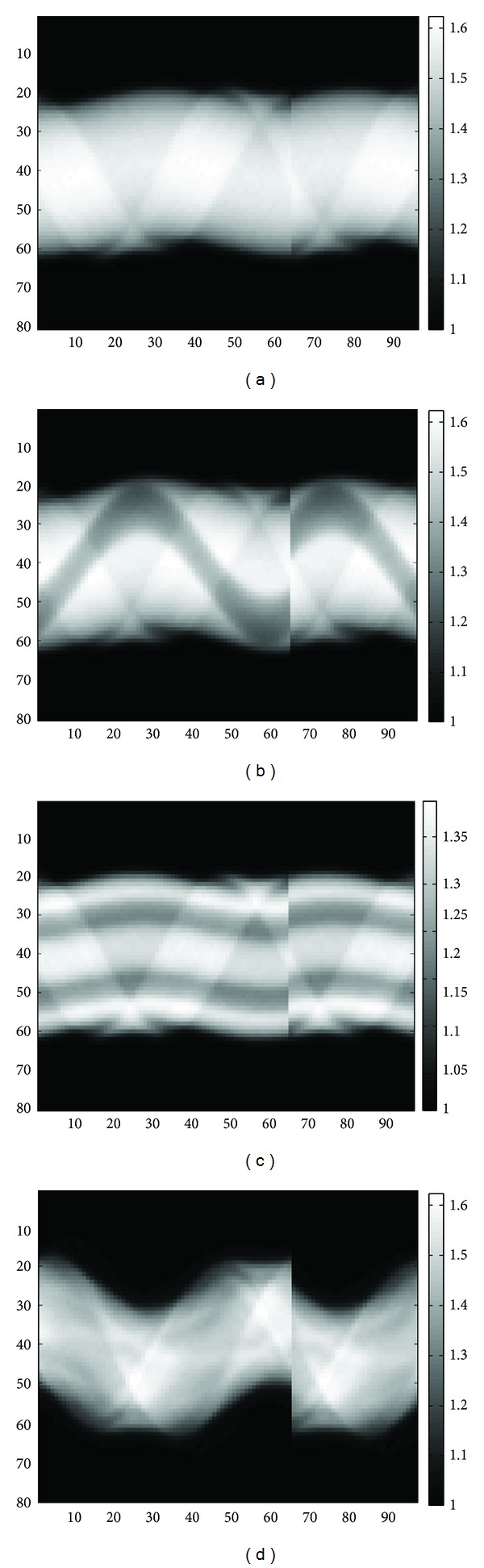
The attenuation correction factor matrix corresponds to the projection data of 41th slice of (a) the rat-sized phantom, (b) the four-quarter phantom, (c) the concentric phantom, and (d) the rat experiment.

After calculation, the AC factor matrix corresponding to the projection data of any slice can be acquired. In Figure 2, the vertical axis corresponds to different positions of detector element, the horizontal axis corresponds to the scanning angles of SPECT, from *θ*_1_ to *θ*_32_ are the AC factor matrixes relative to the projection data of any slice on the head 1 gamma camera, from *θ*_33_ to *θ*_64_ are the AC factor matrixes relative to the projection data of any slice on the head 2 gamma camera, and from *θ*_65_ to *θ*_96_ are the AC factor matrixes relative to the projection data of any slice on the head 3 gamma camera. Because head 1 and head 2 gamma cameras scan from 0° to 180° and 180° to 360°, respectively, and head 3 gamma camera scans from 90° to 270°, those sinograms acquired after imaging and the AC factor matrix calculated by CTMA seem to have a fracture due to the discontinuous imaging angles. However, it will not affect image reconstruction and AC. Numbers 1, 2, 3 are the gamma cameras' codes that system assigns, the codes are not very important, and the important thing is which head covers which projection angles in the imaging process. After the attenuation correction factor matrixes were multiplied by the decay and scatter corrected projection data and combined with FBP to perform image reconstruction, we can acquire the attenuation and scatter corrected SPECT images in this way.

### 2.8. Calibration Factor

The calibration factor (CF) is the ratio of the activity concentration to the voxel value in the reconstructed SPECT image [1, 28]. We used a ^99m^Tc point source for calibration, and the activity of the point source was 41.71 MBq measured by a well-counter. This calibration scan totally took 192 minutes. After decay correction, the images were reconstructed by FBP. Consider

(8) $$\begin{array}{c} \text{CF}=\frac{A}{V\times \text{TC}}, \end{array}$$

where *A* is the activity of the point source measured by a well-counter, *V* is the volume of a voxel, and TC is the summation of voxel value all over the volume-of-interest (VOI), which is 3 cm^3^ including this point source. After scaling by the calibration factor, the voxel value directly represents the activity concentration at this voxel position.

### 2.9. Physical Phantoms and Small Animal Imaging

**Figure 3:**
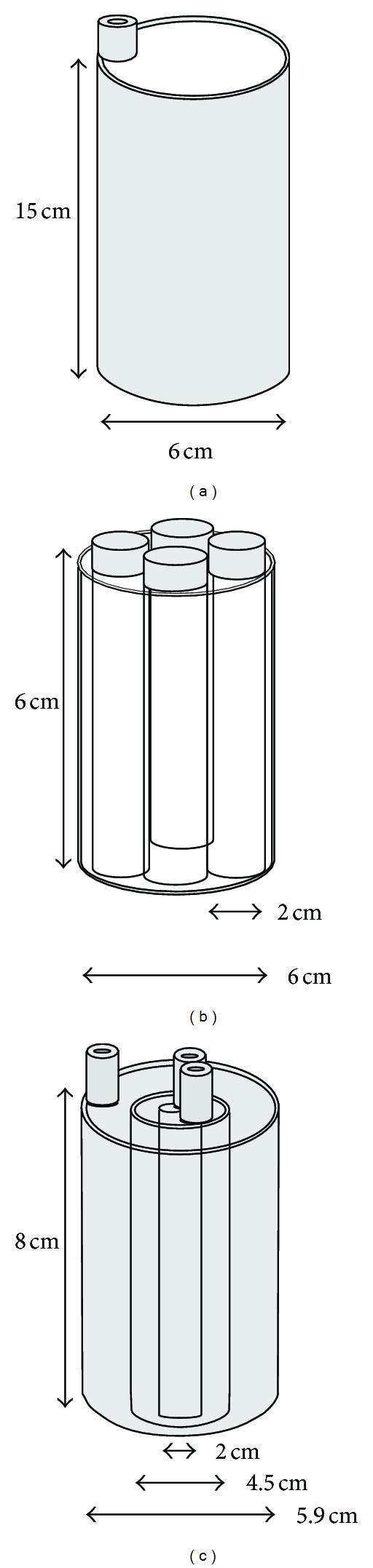
The schematic diagram of (a) the rat-sized phantom, (b) the four-quarter phantom, and (c) the concentric phantom.

Figure 3 shows that the rat-sized phantom is a hollow acrylic cylinder phantom, whose diameter is 6 cm, 15 cm high, 0.2 cm thick, and we filled the phantom with ^99m^Tc solution during the experiment. When we started to scan, the activity concentration was 221.576 MBq. The four-quarter phantom is an acrylic cylinder phantom, whose diameter is 6 cm, 6 cm high, which contains 4 symmetrically located hollow cylinders, whose diameter is 2 cm, 5.8 cm high. We filled the cylinders with air, water, and two different activity concentrations of ^99m^Tc solution during the experiment. When we started to scan, the ^99m^Tc activity concentrations of the two uniform cylinders were 221.576 MBq and 2.417 MBq. The concentric phantom consists of 3 layers of acrylic walls. The diameter of the outside layer is 5.9 cm, the middle layer is 4.5 cm, the inner layer is 2 cm, and the height is 8 cm, while the thicknesses of all walls are 0.2 cm. We filled the phantom with air and two different activity concentrations of ^99m^Tc solution during the experiment. When we started to scan, the activity concentrations from outside to inside were 1.51 MBq, 0 MBq, and 9.4225 MBq. SPECT acquisition time is about one hour each experiment.

As for the animal experiment, we used a 0.4751 kg normal male SD rat for myocardial perfusion imaging. Firstly, ^99m^Tc-sestamibi in 72.853 MBq was injected from the tail vein, and after 40 minutes, data acquisition was performed.

In the imaging process of the physical phantoms and animal experiments, we used totally three gamma cameras equipped with parallel-hole collimators to receive the projection data; each camera scans 180° and takes 32 projection angles.

SPECT used a 5% wide (137 keV to 144 keV) main energy window to scan objects, and afterwards, the iradon.m command in MATLAB 9.0a published by MathWorks was used, which is FBP, combined with Hamming filter to reconstruct images with the scanned projection data. In this way, the object images can be reconstructed in 1.5 × 1.5 × 1.5 mm^3^ voxel size with an 80 × 80 × 80 image matrix. In all physical phantom experiments, we used ^99m^Tc as the tracer. As for the rat experiment, we used ^99m^Tc-sestamibi as the tracer.

CT scans of physical phantoms and a rat were acquired using 80 kVp tube voltage and 90 *μ*A tube current. The gantry rotated in continuous flying mode, a total of 512 projections were acquired in a full 360° scan. Images were reconstructed using modified cone-beam Feldkamp algorithm resulting in 0.17 × 0.17 × 0.17 mm^3^ voxel size with a 512 × 512 × 512 image matrix [35].

### 2.10. Parameter Assessments of Physical Phantom Experiments

As for the physical phantom experiments, the activity concentration inside of phantoms can be obtained by measurement and calculation. By enclosing the VOIs in the same positions, we can evaluate the advantages and disadvantages of the four different attenuation correction methods through arithmetic mean (mean), coefficient of variation (CV), root mean square error (RMSE), normalized root mean square error (NRMSE), and mean percentage error (MPE). Consider

(9) $$\begin{array}{c} \text{Mean}=\frac{1}{n}\sum_{i=1}^{n}a_{i},\\ \text{CV}=\frac{\text{SD}}{\text{Mean}},\\ \text{RMSE}=\sqrt{\frac{1}{n}\sum_{i=1}^{n}{\left(a_{i}-a_{t}\right)}^{2},}\\ \text{NRMSE}=\sqrt{\frac{1}{n\cdot {a_{t}}^{2}}\sum_{i=1}^{n}{\left(a_{i}-a_{t}\right)}^{2},}\\ \text{MPE}=\frac{1}{n}\sum_{i=1}^{n}\frac{a_{i}-a_{t}}{a_{t}}, \end{array}$$

where *n* is the number of pixels in the VOI, *a*_*i*_ is the activity concentration at the *i*th position, and *a*_*t*_ is the true value in the VOI.

The true values are the activity concentrations of physical phantoms and animal experiments while we start to scan.

## 3. Results

### 3.1. Physical Phantom Experiments

**Figure 4:**
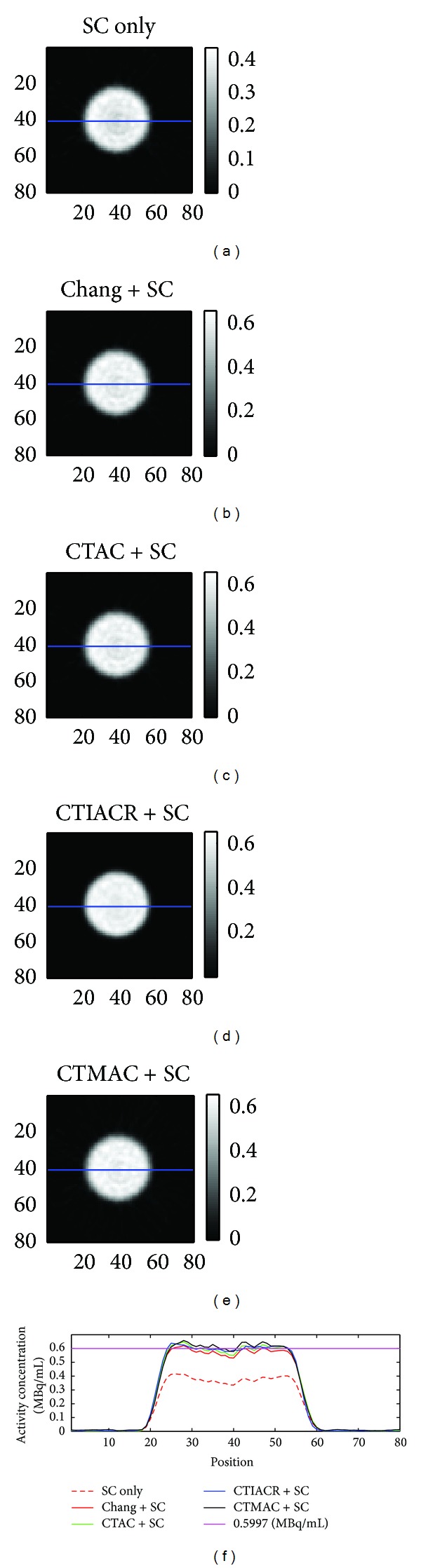
The images of the rat-sized phantom acquired after corrections. (a) Scatter correction, (b) Chang's method in combination with scatter correction, (c) CTAC in combination with scatter correction, (d) CTIACR in combination with scatter correction, and (e) CTMAC in combination with scatter correction. The line profiles through the center of the rat-sized phantom are shown, and the purple line is the true activity concentration obtained from measurement.

Figure 4 is the rat-sized phantom image corrected by different methods. After attenuation correction, the activity per unit volume in the central region of the phantom that was underestimated had flattened out, the overall activity concentration was also pulled to close to the true value, and this is able to reflect the real situation of the homogenous distribution of radionuclide inside the rat-sized phantom.

From Table 1, it can be found that the results corrected with four attenuation correction methods very close to the true activity concentration. If the mean percentage error (MPE) is taken as the figure of merit (FOM), the best correction method is CTAC, and the relatively poor method is CTMAC. Although the results corrected by CTMAC of the rat-sized phantom are relatively poor, it is still a very good correction method since MPE is only 3.8%.

**Figure 5:**
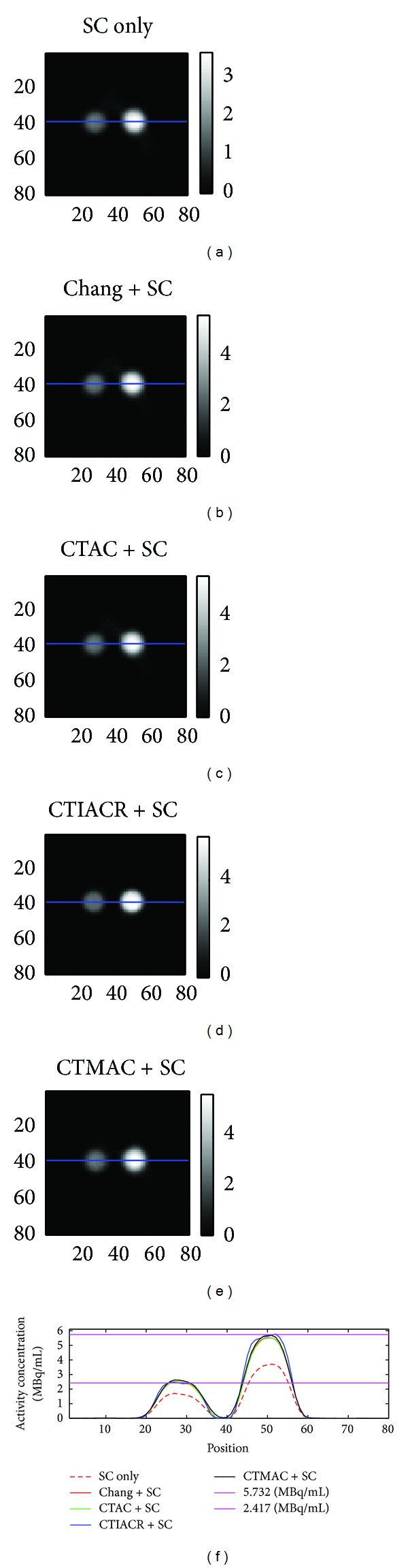
The images of the four-quarter phantom acquired after corrections. (a) Scatter correction, (b) Chang's method in combination with scatter correction, (c) CTAC in combination with scatter correction, (d) CTIACR in combination with scatter correction, and (e) CTMAC in combination with scatter correction. The line profiles through the center of the four-quarter phantom are shown, and the two purple lines are the true activity concentrations obtained from measurements.

Figure 5 is the four-quarter phantom image corrected by different methods. The four hollow cylinders are filled with air, water, and ^99m^Tc solutions in two different activity concentrations; one of which is 2.417 MBq/mL while the other is 5.732 MBq/mL. After attenuation corrections, the activity concentrations of both cylinders filled with ^99m^Tc solutions are corrected to close to true values.

From Table 2, it can be found that the best correction method at the higher activity concentration is CTMAC, and the poorest method is CTAC. Although CTAC is poor, the correction effect is still pretty good since MPE is only 4.35%. At the lower activity concentration, the best correction method is CTIACR, and the relatively poor method is CTMAC. Although the correction result of CTMAC is rather poor, the correction effect is not bad since MPE is only about 7.2%.

**Figure 6:**
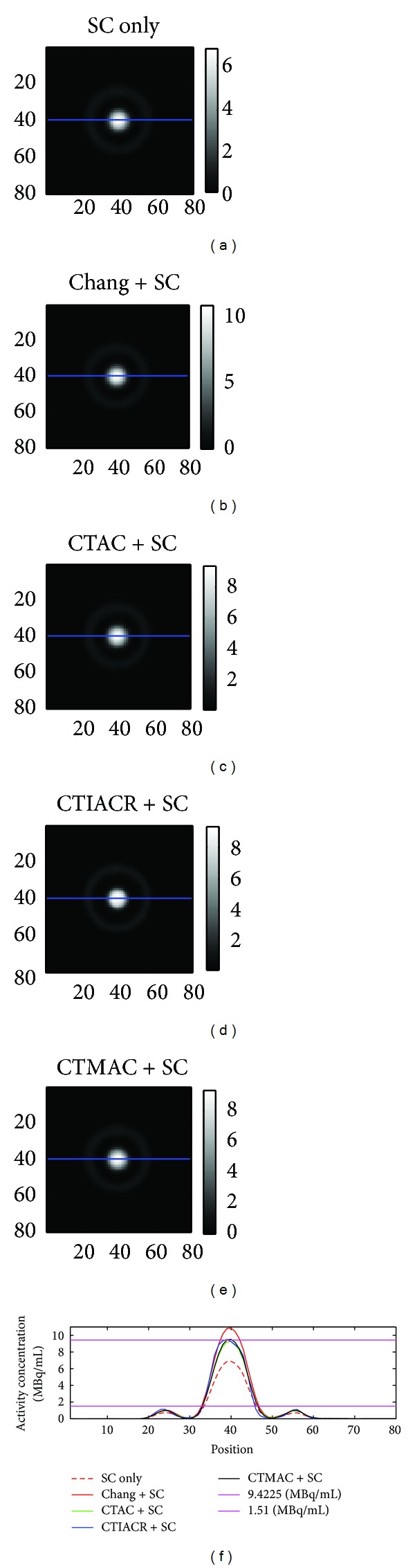
The images of the concentric phantom acquired after corrections. (a) Scatter correction, (b) Chang's method in combination with scatter correction, (c) CTAC in combination with scatter correction, (d) CTIACR in combination with scatter correction, and (e) CTMAC in combination with scatter correction. The line profiles through the center of the concentric phantom are shown, and the purple lines are the true activity concentrations obtained from measurements.

Figure 6 is the concentric phantom image corrected by different methods. The spaces between three concentric layers are filled with air and ^99m^Tc solutions in two different activity concentrations. The activity concentration is 9.4225 MBq/mL in the inner layer and 1.51 MBq/mL in the outer layer. After attenuation corrections, the activity concentrations are all corrected to close to the true values except the image of the inner layer filled with ^99m^Tc solution corrected by Chang's method.

From Table 3, it can be found that the best correction method at the inner layer is CTMAC, and the poorest method is Chang's method. Because Chang's method assumes that the linear attenuation coefficients inside of the contour are the same, thus it will produce higher MPE of 13.407% when the correction is performed on the physical phantoms that have significant differences in the internal structures. As for the outer layer, the best correction method is CTIACR, and the poorest method is CTAC. It can be found that the quantitative results after attenuation, scatter, and decay corrections are still not good because the outer layer is affected more due to the fact that the resolution of the imaging system at center is better than at periphery.

### 3.2. Animal Experiments

**Figure 7:**
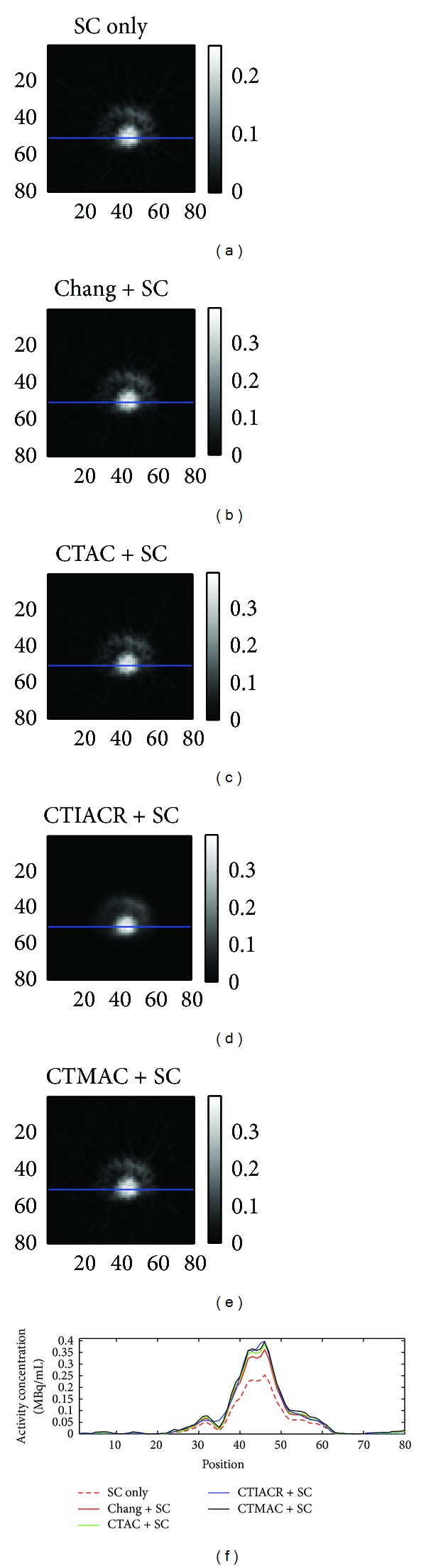
Myocardial perfusion images in a rat acquired after corrections. (a) Scatter correction, (b) Chang's method in combination with scatter correction, (c) CTAC in combination with scatter correction, (d) CTIACR in combination with scatter correction, and (e) CTMAC in combination with scatter correction. The line profiles through the heart center of a rat are shown.

Figure 7 is the myocardial perfusion image of a rat corrected by different methods. After attenuation corrections, in some of the myocardial region, where the amount of ^99m^Tc-sestamibi was originally underestimated, have been increased later; the activity concentration of ^99m^Tc-sestamibi in the heart was about 0.4 MBq/mL, and from the profiles we can also observe that the distribution of activity concentration corrected by CTAC (green contour) and CTMAC (black contour) is very similar.

From the images we can find that the blood and muscle contain unabsorbed ^99m^Tc-sestamibi, the same as the blood in two atria and two ventricles, considering that it will cause the operating area pollution easily and the influence of the unabsorbed ^99m^Tc-sestamibi in blood; the heart of the rat was not taken out to measure the activity concentration. We only observe whether there is significant difference between the distributions of activity concentration corrected by the four attenuation correction methods combining with the scatter correction.

### 3.3. Required Time for Attenuation Corrections in Combination with Scatter Correction

The computer equipment used in this study is described as follows:

- CPU: AMD Phenom (tm) II X3 720 Processor 2.80 GHz;
- RAM: 4 GB;
- System type: 32 bits.

From Table 4 we can know that the time required for TEW method in combination with CTMAC is the shortest, and TEW method in combination with Chang's method or CTAC is longer; however, none of them exceeds eight minutes. The time required for correction will be changed along with the number of subsets and the number of iterations when TEW method is combined with CTIACR, and the correction time required for CTIACR in Table 4 is the correction time of the rat-sized homogeneous phantom which is two iterations with eight subsets; the correction time is shorter than Chang's method and CTAC because CTIACR only performs attenuation compensation in the central region of a 54 × 54 matrix size.

## 4. Discussion

Small-animal SPECT images are typically much less degraded by photon attenuation and scatter than clinical SPECT images because of smaller body dimensions; however, the influences of these effects on quantification still cannot be ignored. Hwang et al. [36] found that the activity concentration will be underestimated about 25% on the imagining result obtained from a 2 cm radius cylinder phantom which is filled with ^99m^Tc solution. For the imaging data obtained from FLEX Triumph preclinical imaging system, it is not necessary to move small animal or phantom during the imaging process; thus image registration and fusion are very precise and easy.

The scattered photons will increase the low frequency components of images and decrease the contrast [14]. Applying TEW method to physical phantom experiments for scatter correction, the quantitative accuracy of activity concentration will be underestimated 9% without any correction to 33% with correction. If attenuation correction is only performed with the acquired data, the distribution of activity concentration converted from corrected images will be overestimated, and these are the wrong correction results due to using the attenuation correction factors calculated by narrow-beam attenuation coefficients to correct the SPECT data in broad-beam conditions [21, 37].

The X-rays emitted from the X-ray tube of CT demonstrate a spectral distribution of multiple energy. In the processes that the beam penetrates object, the low-energy photons are relatively easy to be absorbed; thus the beam hardening artifact will be generated, and after converting by bilinear calibration curve, the linear attenuation coefficients at the thicker region will be smaller than the thinner region [35]. For gamma rays in relatively high energy, the bilinear calibration curve is more accurate for converting CT numbers to linear attenuation coefficients than linear relationship [16]. Brown et al. [24] considered that when the gamma rays energy is lower than 140 keV, using linear relationship to convert CT numbers to linear attenuation coefficients is accurate enough. The transformation effects of lower errors can be apparently observed when the gamma rays energy are between 140 keV and 364 keV by using the bilinear calibration curve; thus using a linear relationship to convert CT numbers to linear attenuation coefficients is enough in this study.

As for the four-quarter phantom, Chang's method is slightly better than CTAC because the four-quarter phantom has more acrylic part, and it is different from the situation that water in the rat-sized phantom occupies most of the volume; thus it is obviously improper when the uniform attenuation maps used in the four-quarter phantom are the same as that used in the rat-sized phantom whose linear attenuation coefficients were given in a situation of 140 keV photons in water, since the density of acrylic is higher than water, and the four hollow cylinders in the four-quarter phantom are filled with air, water, and ^99m^Tc solutions of two different activity concentrations; that is to say, one cylinder has air, and the other three cylinders have solutions. It is possible that the average linear attenuation coefficient of 140 keV gamma rays in the four-quarter phantom is very close to the linear attenuation coefficient of 140 keV gamma rays in water of 0.15454 cm^−1^ by chance; thus a situation appeared that the correction results of Chang's method are better than CTAC. However, in addition to Chang's method, the other methods are all done by CT to acquire nonuniform attenuation maps of object, and thus the attenuation correction results of the four-quarter phantom acquisition data are still valuable for the assessment of the other three attenuation correction methods that use nonuniform attenuation map.

In the processes of experiments, there are two factors that will affect the quantitative results. One is the energy window setting and the other is the well-counter used. Different subwindows settings will cause different results of scatter photon counts in the assessment and further affect quantification. Therefore, in different physical phantom experiments and animal experiment, the same settings for the main energy window and subwindows must be used. If the well-counter is not accurate, the calibration factor and the assessment results of quantitative corrections are also wrong. Thus, an accurate well-counter is indispensable for quantification of activity concentration [1].

The ranking of the results corrected by the four attenuation correction methods on different physical phantoms and the structures of different areas in phantoms may not always be the same since it is related to their own features of the four attenuation correction methods. From the errors after corrections, it can be found that the activity concentration corrected by CTMAC is always slightly higher than CTAC, but the difference is not significant. This situation is related to that when using CTMAC to calculate the average linear attenuation coefficient of any detector element at different projection angles, the linear attenuation coefficients of all pixel positions located on that LOR are considered. The sampling process of linear attenuation coefficients of any attenuation path of any pixel position for CTAC does not consider the linear attenuation coefficient of the starting point pixel even though the starting point has *μ* value, but the length of attenuation path is 0. Certainly, it is considered that a point source less or equal to the pixel size can barely cause photon attenuation. Therefore, the results corrected by CTMAC are always higher than CTAC. Under the circumstance that the results corrected by the both methods are all higher than the true activity concentration, the results corrected by CTMAC have higher errors with respect to the true activity concentrations, for example, the rat-sized phantom and the parts that have lower activity concentration in the four-quarter phantom. Under the circumstance that the results corrected by both methods are lower than the true activity concentration, the results corrected by CTMAC will be closer to the true activity concentration, for example, the parts that have higher activity concentration in the four-quarter phantom and the most inner layer and the most outer layer of the concentric phantom. CTIACR will be affected by the iteration number and the number of subset, and the quantitative results will be different accordingly. We take the corrected results that are closest to the true activity concentration in the processes that we try to change different parameters to display. The errors of Chang's method depend on the difference between the nonuniform attenuation map and the attenuation map actually used. This is related to the structural designs, materials, and filled contents of the physical phantom. The selected size and locations of regions of interest (ROIs) as well as the distance between the structures in different areas and the bed or other shields also have influences (when sampling the linear attenuation coefficients on the photon attenuation paths at different projection angles, the closer the shield to the calculated pixel position, the covered range of projection angles will be larger; the farther the shield to the calculated pixel position, the covered range of projection angles will be smaller). Therefore, the ranking of the quantitative results performed on different physical phantoms and on structures of different areas in phantoms by the four attenuation correction methods will be affected by the calculation processes of the correction methods and their own characteristics of the correction methods. Thus, different rankings were showed.

As CTMAC is a concept of average, it could be wrong if we observed the corrected projection data only in a single direction. For example, assume that a source is exactly behind the attenuator, and using the attenuation correction factor calculated by the average linear attenuation coefficient and the average length of the photons' attenuation paths to correct the measured projection data will cause the corrected projection values lower than the true projection values. However, the projection data acquired on the opposite side are that the attenuator is exactly behind the source. Then the projection values corrected by CTMAC will be higher than the true values. The projection values in the remaining directions corrected by CTMAC are almost equal to the true projection values because there is no blocking attenuator. Since it is an average method, when the scanning angles are sufficiently dense enough and the angular range covered during imaging is sufficiently large by gamma cameras, the corrected projection data reconstructed by FBP will be very close to the distribution of true activity concentration.

The calibration factor is relevant to the factors of photon energy, the type, materials, aperture diameter of collimator, the acquisition time per projection, the number of projections each gamma camera scans, and the radius of gyration of the camera heads and so forth, and there is not much difference between various reconstruction methods; it has also no relationship to hand-made software.

## 5. Conclusions

As for rat-sized objects, the issue that the effect of photon attenuation causes inaccurate quantitative results is worthy to consider. The quantification corrected by CTMAC in combination with scatter correction is the same as those attenuation correction methods that are broadly applied currently, the errors are within the acceptable range, and the time required for correction is shortest. Thus, it has the value for practical applications, and CTMAC can be applied in clinical applications as well. As for the animal experiment, the distribution of activity concentration corrected by CTMAC and CTAC is almost the same. With the data by micro-CT, performing the attenuation corrections in parallel-hole SPECT imaging data in combination with scatter correction can obtain the accurate quantification of activity concentration of radioisotope. 

## Figures and Tables

**Table 1:** Assessing the results after corrections of rat-sized phantom with five different parameters.

	Mean	CV	RMSE	NRMSE	MPE
Original	0.53612	0.03770	0.06675	0.11129	−0.10610
SC only	0.37846	0.05251	0.22217	0.37045	−0.36900
Chang + SC	0.58165	0.04194	0.03035	0.05060	−0.03020
CTAC + SC	0.60398	0.04257	0.02603	0.04340	0.00706
CTIACR + SC	0.60757	0.02789	0.01865	0.03110	0.01305
CTMAC + SC	0.62259	0.03755	0.03267	0.05448	0.03810

The true value of the activity concentration is 0.5997 MBq/mL.

**Table 2:** Assessing the results after corrections of four-quarter phantom with five different parameters.

	Mean	CV	RMSE	NRMSE	MPE
The part of higher activity concentration in 4-quarter phantom					

Original	5.01020	0.00588	0.72227	0.12601	−0.12590
SC only	3.67710	0.00784	2.05510	0.35852	−0.35850
Chang + SC	5.49350	0.00370	0.23920	0.04173	−0.04160
CTAC + SC	5.48270	0.00308	0.24972	0.04357	−0.04350
CTIACR + SC	5.66160	0.01463	0.10222	0.01783	−0.01230
CTMAC + SC	5.66780	0.00352	0.06666	0.01163	−0.01120

The part of lower activity concentration in four-quarter phantom					

Original	2.25120	0.01806	0.17031	0.07047	−0.06860
SC only	1.66000	0.02205	0.75784	0.31355	−0.31320
Chang + SC	2.48390	0.01487	0.07577	0.03135	0.02770
CTAC + SC	2.47640	0.01622	0.07086	0.02932	0.02459
CTIACR + SC	2.42750	0.02335	0.05547	0.02295	0.00436
CTMAC + SC	2.59160	0.01451	0.17826	0.07376	0.07223

The true value of the higher activity concentration is 5.732 MBq/mL.

The true value of the lower activity concentration is 2.417 MBq/mL.

**Table 3:** Assessing the results after corrections of concentric phantom with five different parameters.

	Mean	CV	RMSE	NRMSE	MPE
The most inner layer of the concentric phantom					

Original	9.02450	0.01507	0.41616	0.04417	−0.04220
SC only	6.77330	0.01769	2.65140	0.28139	−0.28120
Chang + SC	10.68580	0.01764	1.27450	0.13526	0.13407
CTAC + SC	9.10310	0.01811	0.35180	0.03734	−0.03390
CTIACR + SC	9.26040	0.03255	0.31458	0.03339	−0.01720
CTMAC + SC	9.34950	0.01750	0.16349	0.01735	−0.00770

The most outer layer of the concentric phantom					

Original	0.96092	0.05688	0.55053	0.36486	−0.36320
SC only	0.70550	0.05687	0.80432	0.53306	−0.53240
Chang + SC	0.98716	0.05907	0.52479	0.34780	−0.34580
CTAC + SC	0.90757	0.05276	0.60310	0.39971	−0.39850
CTIACR + SC	1.09030	0.06928	0.42502	0.28168	−0.27740
CTMAC + SC	0.96426	0.05810	0.54734	0.36275	−0.36090

The true value of the activity concentration in the most inner layer is 9.4225 MBq/cm^3^.

The true value of the activity concentration in the most outer layer is 1.51 MBq/cm^3^.

**Table 4:** Required time for scatter correction in combination with different attenuation correction methods on a particular slice.

Chang's + SC	CTAC + SC	CTIACR + SC	CTMAC + SC
7.9480 minutes	7.8664 minutes	4.6010 minutes	0.1467 seconds

The reconstruction parameters of CTIACR in combination with scatter correction are two iterations and eight subsets.
